# Post-operative functional neurological symptom disorder after anesthesia

**DOI:** 10.17305/bjbms.2020.4646

**Published:** 2020-08

**Authors:** Ryan S. D’Souza, Matthew N. P. Vogt, Edwin H. Rho

**Affiliations:** Department of Anesthesiology and Perioperative Medicine, Mayo Clinic, Rochester, Minnesota, USA

**Keywords:** Functional neurological symptom disorder, anesthesia, peri-operative period, psychogenic coma, psychogenic non-epileptic seizures, conversion paralysis

## Abstract

A rare manifestation during the post-anesthetic period may include the occurrence of functional neurological symptom disorder (FNSD). FNSD is described as neurological symptoms that are not consistently explained by neurological or medical conditions. We report a case series consisting of six patients who underwent a general anesthetic at a tertiary referral hospital and experienced FNSD in the immediate post-anesthetic period. Life-threatening causes were excluded based on benign physical exam findings and knowledge of past history. Five of six cases manifested with FNSD only in the immediate post-operative setting after exposure to anesthesia, and never otherwise experienced these symptoms during their normal daily lives. MEDLINE and Google Scholar were searched through October 2019 using a highly-sensitive search strategy and identified 38 published cases of post-anesthetic FNSD. Meta-analysis of pooled clinical data revealed that a significant proportion of patients were females (86%), reported a history of psychiatric illness (49%), reported a prior history of FNSD (53%), and underwent general anesthesia as the primary anesthetic (93%). The majority of patients were exposed to diagnostic studies (66% received radiographic tests and 52% received electroencephalogram) as well as pharmacologic therapy (57%). While no deaths occurred, many patients had unanticipated admission to the hospital (53%) or to the intensive care unit (25%). These data may help inform the anesthesia literature on presentation, risk factors, and treatment outcomes of FNSD in the context of anesthetic administration. We contemplate whether anesthetic agents may predispose a vulnerable brain to manifest with involuntary motor and sensory control seen in FNSD.

## INTRODUCTION

Emergence and recovery during the immediate post-anesthetic recovery period is a vulnerable and unpredictable stage for every patient. A rare manifestation during this period may include the occurrence of functional neurological symptom disorder (FNSD). According to the American Psychiatric Association Diagnostic and Statistical Manual of Mental Disorders (DSM-5), FNSD is described as neurological symptoms that are not consistently explained by neurological or medical conditions [1]. Specific examples of FNSD include psychogenic non-epileptic seizures (PNES), psychogenic coma, conversion paralysis, functional movement disorder, blindness, and non-dermatomal sensory deficits [1,2]. Our case series and review will focus on PNES, psychogenic coma, and conversion paralysis.

PNES is often referred to as pseudoseizures and manifests with neurological symptoms similar to an epileptic seizure, although these episodes are not related to abnormal brain electrical activity [3]. The overall prevalence of PNES is between 1/3000 and 1/50,000, and no estimates are reported in the peri-operative setting [4]. Symptoms include abnormal body movements that are waxing and waning typically for a prolonged duration, closed eyes with resistance to eye-opening, positive response to noxious stimuli, and gradual onset of symptoms with abrupt recovery [5]. Releasing the patient’s arm over the face will typically show purposeful arm movement by the patient to protect the face [6]. There is resistance to anti-epileptic medication and after organic causes for seizures have been excluded, treatment is primarily psychiatric care including cognitive behavioral therapy (CBT) and potentially adjunctive medications. The effectiveness of CBT varies widely among patients with PNES [7].

Post-operative psychogenic coma manifests with a prolonged period of unresponsiveness without an organic cause [8]. However, it is paramount that the provider should also evaluate and rule out other devastating causes of delayed awakening from anesthesia including cerebrovascular accident, intracranial hemorrhage, metabolic derangements, anesthetic overdose, and inadvertent drug misadministration. Along the same spectrum, conversion paralysis is a psychiatric disorder with symptoms involving motor or sensory function impairment that cannot be attributed to a neurological condition or other medical condition [9].

These atypical post-anesthetic manifestations remain an obscure topic in the peri-operative setting, and early diagnosis may help prevent iatrogenic injury [6]. The use of hypnotics and anticonvulsants may hinder diagnosis, prolong anesthetic recovery, and importantly introduce potential side effects from unnecessary medications [10]. Furthermore, these symptoms may lead to unanticipated admission to the hospital or intensive care unit (ICU). In here, we described six patients who experienced FNSD following a general anesthetic at a tertiary referral hospital (Mayo Clinic, Rochester, MN). We also performed a systematic review of the literature on papers reporting post-operative FNSD, specifically PNES, episodes of unresponsiveness, or conversion paralysis, in patients after receiving an anesthetic. These data may help inform the anesthesia literature on presentation, risk factors, management, and treatment outcomes of post-operative FNSD in the context of anesthetic administration, and may also facilitate the stratification of patients who are at high-risks for experiencing these spells.

## MATERIALS AND METHODS

This study was reviewed by the Mayo Clinic Institutional Review Board and was deemed exempt (IRB #19-004713). This was a case series consisting of six patients who underwent a general anesthetic at a tertiary referral hospital (Mayo Clinic, Rochester, MN) and experienced FNSD in the immediate post-operative period. We excluded studies reporting these symptoms occurring after 24 hours post-anesthesia. Data on clinical presentation, treatment, and outcomes were captured and described in a de-identified fashion (Table 1).

**TABLE 1:**
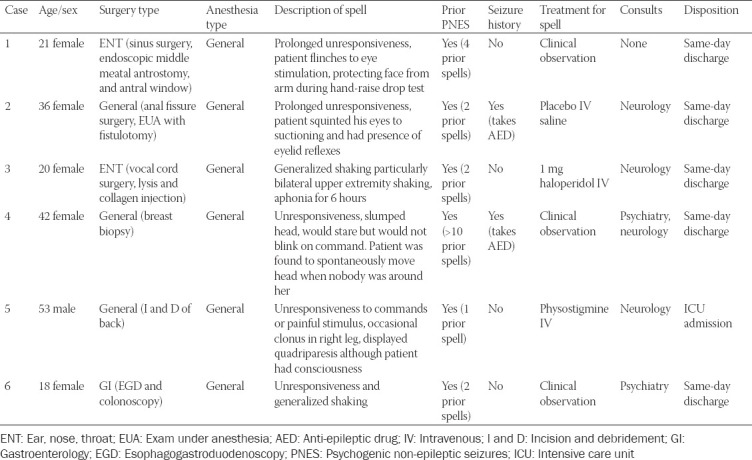
Description of functional neurological symptom disorder episodes in case series

The MEDLINE and Google Scholar databases were searched through September 9, 2019 using a highly-sensitive text word search strategy to find any reports, case series, and observational studies describing other cases of post-operative PNES, psychogenic coma, and conversion paralysis in the immediate post-operative period. Serial searches included the terms “pseudoseizure,” “psychogenic non-epileptic seizure,” “psychogenic coma,” “conversion disorder,” “conversion paralysis,” “psychophysiologic disorder,” “somatoform disorder,” “functional neurological symptom disorder,” “surgery,” “postoperative,” “anesthesia,” and “anesthesia recovery period” independently and in combination using Boolean operators. Specific outcomes addressed included demographic data, past medical history, history of psychiatric illness, description and duration of spell, type of surgery and anesthetic, diagnostic studies, consultations or referrals to psychiatry and/or neurology, treatment administered for spell, and patient disposition. Psychiatric illness was defined as an axis I illness comprising major depressive disorder, generalized anxiety disorder, bipolar disorder, conversion disorder, or alcoholism [8].

## RESULTS

### Case 1 (psychogenic coma)

A 21-year old Iranian female with a history of hypothyroidism, recurrent sinus infections, and dermatographism underwent left sinus surgery under general anesthesia induced with intravenous (IV) propofol, fentanyl, and mivacurium and maintained with sevoflurane and isoflurane. She received a total of 100 mcg IV fentanyl during the case. During emergence, the patient was gagging but did not display purposeful movements and did not follow any commands. She had four robust twitches on train-of-four (TOF) monitor. She was sent intubated to the post-anesthesia care unit (PACU) and was extubated after an hour, although the patient still did not respond to any commands. Neurological exam revealed an intact pupillary response, and it was noted that the patient would flinch her eyes when bright light was shined toward her eyes. Notably, she had a positive hand-raise drop test. She did not respond to noxious stimuli including cold water application to her ears, but did move when a cold hand was placed on her back. After 90 minutes of no improvement, her family was allowed to visit her in the PACU, and then she began to open her eyes and interact with them. The patient reported that she was aware and recalled all events that transpired since extubation. On further discussion, the patient described four similar episodes of unresponsiveness after general anesthesia in Iran. After brief observation in the PACU, the patient was uneventfully discharged.

### Case 2 (psychogenic coma)

A 36-year old female with a history of seizures controlled with phenobarbital, depression controlled with venlafaxine, anxiety, paroxysmal nocturnal dyspnea, postural orthostatic syndrome, and rectal fistula underwent an anal fissure surgery and fistulotomy under general anesthesia induced with IV propofol, lidocaine, and morphine and maintained with sevoflurane and vecuronium. She received a total of 4 mg IV morphine during the case. At the conclusion of the case, she was appropriately reversed with four robust twitches on TOF and was uneventfully extubated. After extubation, she was found to be weak and unresponsiveness for about 90 minutes. Her vital signs remained stable during this time, she was spontaneously breathing, and her pupillary light reflex was intact. Noxious stimuli including suctioning of mouth and insertion of nasal trumpet caused the patient to squint. After 30 minutes of no improvement, her anesthesiologist administered a placebo medication (1 mL of normal saline) into the patient’s IV line. Within 1 minute, she opened her eyes to command and was able to move her extremities to command. An electroencephalogram (EEG) was unremarkable. Upon further chart review, it was noted that she had experienced two similar episodes of psychogenic coma in the past after an atrioventricular node ablation procedure and during an EEG monitoring test. After brief observation in the PACU, the patient was uneventfully discharged.

### Case 3 (PNES)

A 20-year old female with a history of migraines, generalized fatigue, and severe dysphonia from vocal cord problems underwent vocal cord surgery with collagen injection under general anesthesia induced with IV propofol and fentanyl, and maintained under total IV anesthesia (TIVA) with propofol. Jet ventilation was performed during the case. She received a total of 150 mcg IV fentanyl during the case. The case was uneventful and the patient was transferred to the PACU after she displayed spontaneous respirations. In the PACU, she displayed unresponsiveness, hyperventilation, and generalized body shaking that was more prominent in her upper extremities bilaterally and intermittently for a duration of 6 hours. Her vital signs remained stable and she continued to have a patent airway. Due to her hyperventilation, a brown paper bag was placed over the patient’s mouth while she was breathing. After 30 minutes of continued hyperventilation and generalized shaking, 1 mg of IV haloperidol was administered. Ten minutes later, the patient was responsive, following commands, and appropriately verbalizing. Further chart review revealed that the patient had experienced two prior PNES episodes after vocal cord surgeries with an uneventful recovery. She was uneventfully discharged the same day.

### Case 4 (psychogenic coma)

A 42-year old female with a history of seizures treated with lamotrigine, narcolepsy, depression treated with citalopram, and celiac sprue underwent a breast biopsy under general anesthesia induced with IV propofol and fentanyl and maintained with desflurane and nitrous oxide. She received a total of 250 mcg IV fentanyl during the case. After completion of the case, she was noted to have purposeful movement and thus she was extubated. After extubation, she was unresponsive and did not follow commands. She forcibly resisted eye opening and was noted by the nurses to spontaneously move when nobody was in the room with the patient. An EEG performed in the PACU was unremarkable. These symptoms continued for 2 hours until spontaneous resolution, after which the patient reported she was doing well and did not recall any of the events since extubation. Of note, she reported significant psychosocial stressors in her life. Chart review revealed multiple prior spells of psychogenic coma, with over ten documented prior episodes leading to several emergency department visits. She also reported a history of seizure disorder, stating she is diagnosed with “visual epilepsy” and takes lamotrigine. She was uneventfully discharged the same day.

### Case 5 (PNES)

A 53-year old male with a history of chronic back pain status post-lumbar laminectomy and celiac disease underwent an incision and debridement of a back wound under general anesthesia induced with IV propofol, fentanyl, and cisatracurium and maintained with sevoflurane and nitrous oxide. He received a total of 100 mcg IV fentanyl during the case. At the end of the surgery, he was appropriately reversed with neostigmine and glycopyrrolate, but would not respond to verbal or painful stimuli. He was transferred to the PACU, where he continued to remain unresponsive and occasionally displayed clonus in his right leg. He was administered 2 mg of IV physostigmine with no change in neurological status. Serological studies including arterial blood gas, electrolytes, and glucose were normal. After 2 hours, the patient was slowly able to open his eyes and barely raise his thumb to command. Due to concern for locked-in syndrome, neurology was consulted immediately and he was transferred to the ICU. An EEG was performed which was unremarkable even when the patient experienced right lower extremity clonus. Computed tomography head and angiogram were also negative. Symptoms persisted overnight, however, the patient was noted to have a positive hand-raise drop test. He slowly recovered after 2 days with a normal neurological exam back to baseline with no deficits. On direct encounter, the patient reported a prior episode of speaking incomprehensible words for 5 hours after a surgery under general anesthesia. He was thereafter transferred to the floor and was discharged the following day.

### Case 6 (PNES)

An 18-year old female with a history of congenital hip dysplasia, irritable bowel syndrome treated with amitriptyline, and known prior post-operative PNES spells underwent an EGD and colonoscopy under general anesthesia, both induced and maintained with sevoflurane only. At the end of the procedure, she was successfully extubated after return of spontaneous ventilation and regaining consciousness. However, she subsequently displayed generalized shaking and unresponsiveness. During this episode, she had a retained pupillary response to light and no gross neurological deficits. Notably, the anesthesiologist was aware that she had a history of two prior PNES episodes after a hip hardware removal surgery and cholecystectomy surgery, after which EEG was unremarkable. Given this history, the surgery and anesthesia team decided to observe the patient in PACU instead of pursuing additional diagnostic workup. She did not experience any hemodynamic instability or respiratory abnormalities during this spell. After 45 minutes, her symptoms resolved and she was able to verbalize and follow commands. A psychiatry referral was placed and the patient was discharged the same day. Evaluation from the psychiatrist was unremarkable and confirmed a diagnosis of functional spells.

## DISCUSSION

Seizure-like activity, unresponsiveness, and new-onset paralysis are some of the most worrisome neurological manifestations in the immediate post-operative period. We reported six cases encompassing PNES or psychogenic coma after receiving general anesthesia. Importantly, life-threatening causes for symptoms were excluded promptly in every case based on benign physical exam findings and knowledge of pertinent past medical history. In most of our cases, we avoided further tests and procedures such as wide-ranging serological studies, radiographic tests, and EEG. Prompt recognition of PNES, psychogenic coma, or conversion paralysis may prevent unnecessary diagnostic studies and invasive procedures and their associated procedural risks, but it is often beneficial to obtain basic serological studies (complete blood count and basic metabolic panel), EEG, and neurology consult.

Collectively, PNES, psychogenic coma, and conversion paralysis in the immediate post-operative setting are rare, with only few isolated case reports and case series reported in the literature. Furthermore, these diagnoses may have alternate medical names, further compounding the rare presentation and delaying prompt recognition. For example, terms referring to PNES include pseudoseizure, hysteria, and psychogenic non-epileptic episodes. Similarly, psychogenic coma may also be known as conversion coma, dissociative stupor, hysterical coma, and hysterical unconsciousness [8].

Our search strategy identified 38 previously reported cases of PNES, psychogenic coma, or conversion paralysis in the immediate post-anesthesia period [6,8,10-34]. The largest case series was published by Reuber et al. in 2000 that described six cases of post-operative PNES that had been misdiagnosed previously with epilepsy and treated chronically with anticonvulsants [10]. Interestingly, even a case of post-operative PNES has been reported in the peripartum period [13] and in the pediatric population [31]. Authors from these previously published case reports recommend that PNES should be highly considered on the differential in patients with histories of multiple episodes of post-operative seizures. These patients are likely not having their psychiatric needs addressed and may be at increased risk for suicide [10].

Regardless, there is still considerable uncertainty in the literature regarding the etiology of FNSD. Traditionally, FNSD was described as a physical manifestation of psychological distress [2]. Yet, there is limited empirical evidence to support this explanation, and patients may respond negatively to the explanation of a psychiatric cause for their symptoms [35]. Furthermore, while prior research showed that rates of trauma, stress, and psychiatric diseases were higher in patients with FNSD, recent research reveals a low incidence of psychiatric diagnoses in this patient population [36,37]. In addition, neurobiological etiological models have been described for FNSD symptoms [38,39]. A possible explanation may be that patients with FNSD have a decreased sense of control over their actions. For instance, a study comparing patients with FNSD displaying functional tremors versus control patients mimicking tremors demonstrated that there was right temporoparietal junction hypoactivity and decreased functional connectivity between the right temporoparietal junction, limbic region, and sensorimotor cortex [2,40-42]. This suggests that symptoms in FNSD may be perceived to be involuntary even though voluntary motor pathways are being utilized [2]. Notably, 5 of our 6 cases manifested with FNSD only in the immediate post-operative setting after exposure to anesthesia, and never otherwise experienced these symptoms during their normal daily lives at home. We contemplate whether anesthetic agents may predispose a vulnerable brain to manifest with involuntary motor and sensory control seen in FNSD.

Demographic variables and clinical outcome data are displayed in Tables 2 and 3, and in supplemental material. Comparing previously published case reports to our current case series revealed notable similarities that may help the provider recognize this diagnosis. The majority of patients were females (5 of 6, 83% in our cohort; 33 of 38, 87% in published cases) and a high percentage of patients reported a history of psychiatric illness (2 of 6, 33% in our cohort; 18 of 35, 51.4% in published cases), reported a prior history of psychogenic post-anesthetic spell (6 of 6, 100% in our cohort; 15 of 34, 44.1% in published cases), and underwent general anesthesia as their primary anesthetic (6 of 6, 100% in out cohort; 35 of 38, 92% in published cases).

**TABLE 2:**
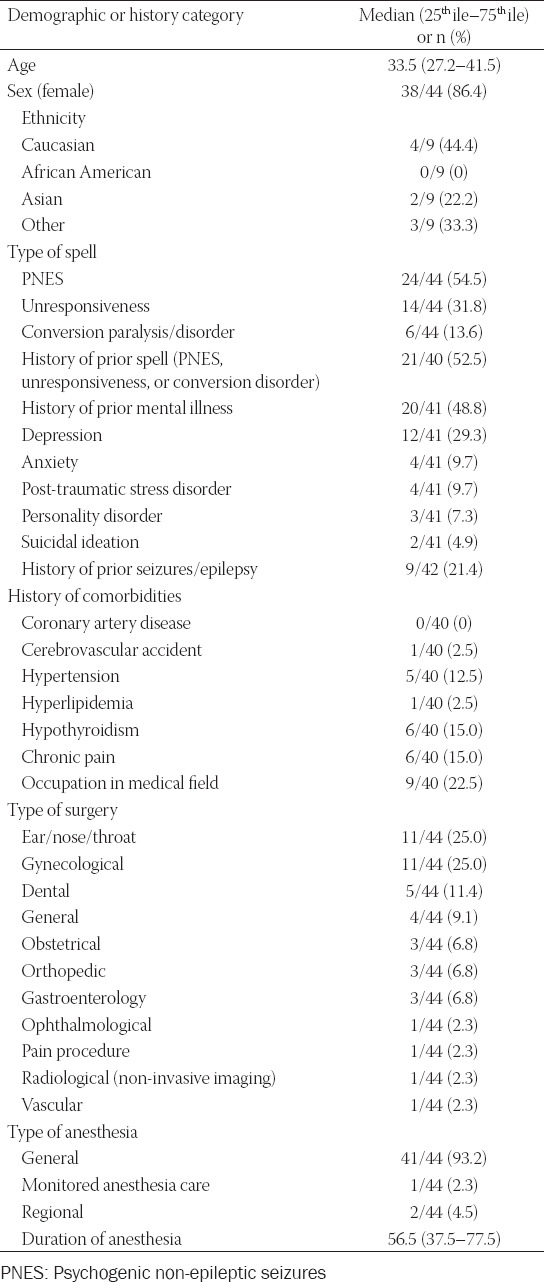
Demographic and clinical history of case series and published cases

**TABLE 3:**
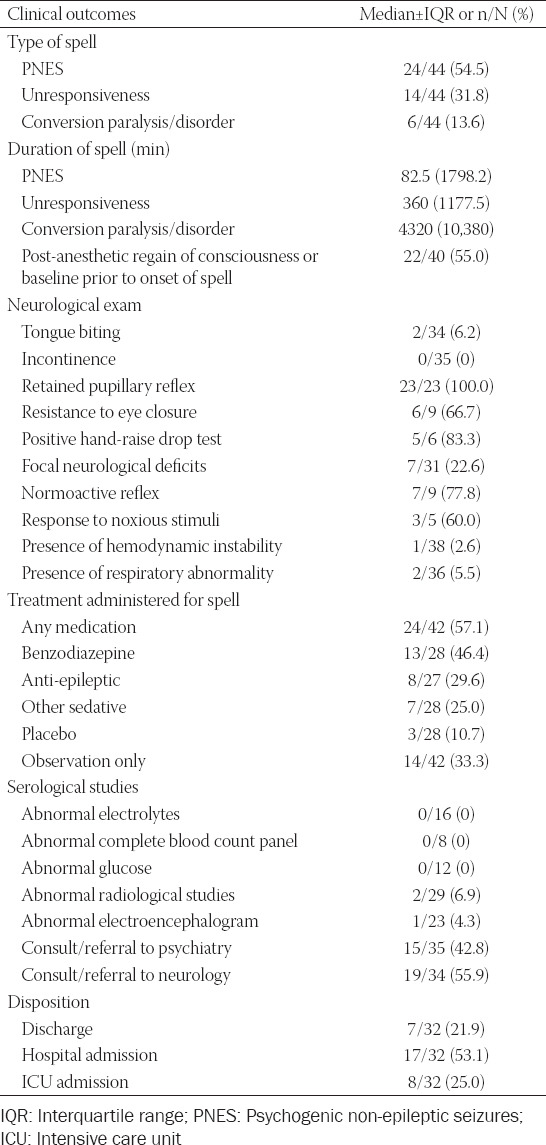
Clinical presentation and outcome of functional neurological symptom disorder episode in case series and published cases

Most cases involved head and neck surgery (2 of 6, 33% in our cohort; 15 of 38, 39% in published cases), either as ear, nose, throat (ENT) cases, dental cases, or ophthalmological cases. Several patients were employed in the medical field (2 of 6, 33% in our cohort; 7 of 34, 21% in published cases). Notably, many patients had a latent period of normal neurological function post-anesthesia prior to the manifestation of their psychogenic spell (1 of 6, 17% in our cohort; 21 of 34, 62% in published cases).

Certain physical exam maneuvers may be utilized to suggest psychogenic etiology of symptoms. A positive “forced eyelid test”, referring to when patients tightly shut their eyelids and resist attempts to open them, was present in 6 of 9 patients (67%). A positive hand-raise drop test, referring to when patients avoid hitting themselves when their arm is raised by a provider and then released, was present in 5 of 6 patients (83%).

The majority of patients were exposed to diagnostic studies, including radiographic imaging tests (66%) and EEG (52%), as well as pharmacologic therapy to treat the spell (57%). Currently, there is no evidence that any long-term medication is useful to treat FNSD and it is generally advised that chronic, long-term pharmacological treatment should be avoided due to associated side effects [43]. Depending on the anesthetic course, it is reasonable to administer naloxone or other opioid reversal agents for suspicion of opioid overdose, flumazenil for benzodiazepine overdose, and physostigmine for the possibility of central anticholinergic syndrome and sleep paralysis [44-47]. Notable medication-related side effects in our case series included unanticipated tracheal intubation due to sedation and respiratory depression from parenteral diazepam, chlormethiazole, thiopentone, and alfentanil in one patient [17], and unresponsiveness in an another patient with hypercapnia (arterial blood gas pH of 7.15 and pCO_2_ of 65 mm Hg) after administration of 2 mg IV midazolam [11]. In another 22-year old otherwise healthy patient with post-operative conversion paralysis of his left-sided extremities, stroke protocol and treatment were initiated for suspicion of brain infarction [32]. Two patients were also exposed to unanticipated invasive spine surgery as a result of their conversion paralysis mimicking spinal cord pathology: a 37-year old male status-post left laminotomy and L5-S1 diskectomy who experienced conversion paralysis with left-sided lower extremity weakness and subsequently underwent re-exploration of the L5-S1 disc space [18], and a 45-year old female status-post C6-C7 arthroplasty who experienced conversion paralysis with complete left-sided hemiplegia only sparing the face and subsequently underwent re-exploration of the C6-C7 disc space [30].

No deaths were experienced in our cohort and in all published cases. However, the majority of patients had unanticipated admission either to the hospital (53%) or to the ICU (25%). Only 22% of patients were discharged the same day. After exclusion of life-threatening causes and diagnosis of a psychogenic etiology for patient’s symptoms, supportive care is primarily recommended with limited diagnostic testing and invasive tests. Consideration of a psychiatry consult or referral is highly recommended to evaluate the patient for an underlying psychiatric illness; this was an under-utilized modality as only 43% of cases underwent evaluation by a psychiatrist after experiencing their post-anesthetic spell. Studies demonstrate that CBT may be beneficial in the treatment of FNSD, and involves educating the patient about the stress response cycle in FNSD, training the patient with behavioral skills and techniques in stress management, and helping patients change harmful and negative thought patterns that reinforce their FNSD symptoms [48].

Future larger-scale observational studies are warranted to further identify risk factors, optimal management, and prognosis in this unique population of patients. Additionally, we defined certain outcomes (e.g prior psychiatric history) as binary variables in our primary analysis; it would be useful to see if chronicity of certain risk factors over time is associated with more post-operative PNES, psychogenic coma, or conversion paralysis.

Our case series and systematic review should be interpreted in the context of multiple limitations. While our size of six cases equates to the largest published case series, future larger-scale observational studies would be beneficial to describe this rare post-operative phenomenon. Given the retrospective nature of the study, there were several missing data points for cases across multiple key variables. We also did not abstract data on the chronicity of certain risk factors (e.g duration of prior psychiatric illness, number of prior post-operative psychogenic spells); this is likely a factor of our institution being a tertiary-referral center where many patients were referred for surgery from an outside institution.

## CONCLUSION

PNES, psychogenic coma, and conversion paralysis are an uncommon manifestation in the immediate post-anesthetic period. High suspicion should be given to this diagnosis after excluding life-threatening causes and when physical exam signs are inconsistent with an organic cause, particularly in the presence of risk factors. Potential risk factors include female sex, history of prior psychogenic post-anesthetic spell, psychiatric illness, general anesthesia, and head/neck surgery. Prompt diagnosis and management of this condition can prevent unnecessary diagnostic studies, invasive procedures and their associated potential complications, and hospital cost.
